# Are accessory hearing structures linked to inner ear morphology? Insights from 3D orientation patterns of ciliary bundles in three cichlid species

**DOI:** 10.1186/1742-9994-11-25

**Published:** 2014-03-19

**Authors:** Tanja Schulz-Mirbach, Friedrich Ladich, Martin Plath, Brian D Metscher, Martin Heß

**Affiliations:** 1 Department Biology II, Zoology, Ludwig-Maximilians-University, Martinsried, Germany; 2 Department of Behavioral Biology, University of Vienna, Vienna, Austria; 3 J.W. Goethe-University Frankfurt am Main, Evolutionary Ecology Group, Frankfurt am Main, Germany; 4 Department of Theoretical Biology, University of Vienna, Vienna, Austria

**Keywords:** Hearing enhancement, Macula, 3D orientation pattern of ciliary bundles, Otolith, Interactive 3D models

## Abstract

**Background:**

Cichlid fishes show considerable diversity in swim bladder morphology. In members of the subfamily Etroplinae, the connection between anterior swim bladder extensions and the inner ears enhances sound transmission and translates into an improved hearing ability. We tested the hypothesis that those swim bladder modifications coincide with differences in inner ear morphology and thus compared *Steatocranus tinanti* (vestigial swim bladder), *Hemichromis guttatus* (large swim bladder without extensions), and *Etroplus maculatus* (intimate connection between swim bladder and inner ears).

**Methodology and results:**

We applied immunostaining together with confocal imaging and scanning electron microscopy for the investigation of sensory epithelia, and high-resolution, contrast-enhanced microCT imaging for characterizing inner ears in 3D, and evaluated otolith dimensions. Compared to *S. tinanti* and *H. guttatus*, inner ears of *E. maculatus* showed an enlargement of all three maculae, and a particularly large lacinia of the macula utriculi. While our analysis of orientation patterns of ciliary bundles on the three macula types using artificially flattened maculae uncovered rather similar orientation patterns of ciliary bundles, interspecific differences became apparent when illustrating the orientation patterns on the 3D models of the maculae: differences in the shape and curvature of the lacinia of the macula utriculi, and the anterior arm of the macula lagenae resulted in an altered arrangement of ciliary bundles.

**Conclusions:**

Our results imply that improved audition in *E. maculatus* is associated not only with swim bladder modifications but also with altered inner ear morphology. However, not all modifications in *E. maculatus* could be connected to enhanced auditory abilities, and so a potential improvement of the vestibular sense, among others, also needs to be considered. Our study highlights the value of analyzing orientation patterns of ciliary bundles in their intact 3D context in studies of inner ear morphology and physiology.

## Background

Modern bony fishes (Teleostei) show a high diversity of inner ear morphology and auditory capabilities such as auditory sensitivities and detectable frequency ranges (for an overview see [1-4]). This structural and functional diversity far exceeds that observed in amniotes and renders teleost fishes an interesting group to study adaptations to different environments from the bioacoustical perspective (e.g., [5,6]). However, the underlying selection forces on (and constraints to) the diversification of teleost inner ears and hearing abilities (e.g., [2,6,7]), as well as the relationships between differences in inner ear morphology and function, are still poorly understood [6-8]. One important morphological adaptation is the connection (or close proximity) of a gas-filled compartment—such as the swim bladder—to the inner ears [9]. Gas-filled bladders within a sound field respond to sound pressure changes by compression and expansion and subsequently by oscillations of the (swim) bladder resulting in near field particle motion detection. This enables fishes to perceive sound pressure changes in the far field and results in enhanced auditory abilities as compared to species lacking ancillary auditory structures [2,10]. The general question arises whether such modifications of ancillary auditory structures (e.g. of the swim bladder) and hearing improvements come along with structural modifications of the inner ears.

The inner ear of teleosts consists of three semicircular canals and three otolithic end organs, namely the utricle, saccule and lagena [11]. In teleosts, the hair cells of the sensory epithelia of the otolithic end organs (maculae) are stimulated by particle motion caused by a sound source [11]. The massive calcium carbonate biomineralisate (otolith) overlying each macula is about three times denser than the surrounding tissue; particle motion emanating from a sound source leads to a lagged movement of the otolith relative to the macula, resulting in shearing movement of the ciliary bundles of the sensory hair cells [8,11,12]. Deflection of the bundle towards the eccentrically placed kinocilium provokes stimulation of the hair cell (e.g., [13-15]). Within the macula, the morphologically and physiologically polarized hair cells are organized in different orientation groups of ciliary bundles, and those differences in orientation are thought to play an important role in sound source localization (e.g., [16]).

Studies investigating the maculae of teleost inner ears have suggested that modifications of the swim bladder and accompanying improvements of auditory abilities may be coupled with modifications of the orientation patterns of ciliary bundles of hair cells, mainly in the sensory epithelium of the saccule (macula sacculi; for an overview see [8,17,18]). In species without ancillary auditory structures such as swim bladder extensions to the inner ears, the macula sacculi generally shows a “standard” pattern consisting of four orientation groups: two horizontal orientation groups in the anterior part and two vertical ones in the posterior portion [18]. Species that have a close relationship between the swim bladder and the saccule often display deviations from this pattern in that ciliary bundle orientation is either more complex (e.g., in the holocentrid *Myripristis* sp. [19]), or simpler, comprising only two different orientation groups (otophysans, mormyrids [8,18]). Based on the former observation, conclusions on potentially improved auditory abilities have been drawn from the observed complexity of orientation patterns in some deep-sea fishes, along with their swim bladder specializations (e.g., [20,21]). Deep-sea fishes, however, cannot be maintained under standard laboratory conditions, precluding an investigation of their auditory abilities to test this assumption. A comparative study on the perciform family Sciaenidae found that some species with anterior swim bladder extensions show the “dual” orientation pattern [22] that is similar to the standard pattern but with two additional horizontal groups of ciliary bundles in the posterior-most portion of the macula sacculi [18]. In contrast, another sciaenid species (*Bairdiella chrysoura*) with a close inner ear-swim bladder relationship displays a modified, more complex orientation pattern on the macula sacculi [23]. Chaetodontid fishes with and without anterior swim bladder extensions, on the other hand, show similar inner ear morphology and no differences in ciliary bundle orientation patterns [24].

These findings formed the starting point for our present study in which we investigated inner ear morphology and orientation patterns of ciliary bundles in three species of the diverse family Cichlidae (Perciformes), the members of which differ significantly in auditory abilities and swim bladder morphology [25]. The rheophilic, bottom-dwelling slender lion head (*Steatocranus tinanti*; Pseudocrenilabrinae) has a small swim bladder without contact to the inner ears and can only detect frequencies up to 0.7 kHz. The jewel cichlid (*Hemichromis guttatus*; Pseudocrenilabrinae), in contrast, has a large swim bladder that does not contact the inner ears and detects frequencies up to 3 kHz. Finally, the orange chromide (*Etroplus maculatus*; Etroplinae) displays a large swim bladder with swim bladder extensions connecting to the inner ears, and this species is distinctly more sensitive at frequencies between 0.5 and 3 kHz [25,26]. Each of these extensions has a gas-filled part contacting the lagena and a second, pad-like structure that comes close to the posterior and horizontal semicircular canals, and to a recessus connected to the utricle. Diversity of swim bladder morphology and auditory abilities among those cichlids provides an excellent opportunity to ask whether an intimate swim bladder-inner ear connection comes along with a modification of inner ear morphology, in particular with respect to the maculae. Specifically, improved auditory sensitivities in *E. maculatus*, along with the intimate connection between swim bladder and inner ears (especially with the lagena), could be accompanied by distinct changes in inner ear morphology like modified orientation patterns of ciliary bundles of the maculae, especially on the macula lagenae and macula sacculi. Following previous studies on the inner ear morphology of other species with a tight swim bladder-inner ear relationship (e.g., [22,27]), we predicted to find a larger anterior region of the macula sacculi in *E. maculatus*[6], a higher density of ciliary bundles on the macula lagenae and the macula sacculi, and larger, heavier lagenar and saccular otoliths.

We further asked whether a reduction of the swim bladder as an adaptation to a rheophilic lifestyle (*S. tinanti*), which is associated with poorer auditory abilities [25], also correlates with a changed inner ear morphology, e.g., in form of a reduction of the complete inner ear, smaller maculae or decreased otolith size (see [28]). Based on a recent methodological study [29], we compared orientation patterns of ciliary bundles among those three species according to the three-dimensional curvature of the respective macula. This allows, for the first time, a detailed interpretation of potential inter-specific differences in orientation patterns of ciliary bundles not only on artificially flattened maculae, but while considering the natural 3D curvature of these sensory epithelia in their intact morphological context.

## Material and methods

### Study animals

Test subjects originated from local fish suppliers and were transferred to the University of Vienna in August/September 2011, January/August 2012, and February 2013. Animals were kept separated by species in 98- or 245-l aquaria, which were equipped with a sand bottom, and halved flower pots as hiding places. Fishes were kept under a 12:12 h L:D cycle at 25 ± 1°C and were fed once daily with commercial flake food and chironomid larvae or brine shrimps.

For all investigations, fish were sacrificed according to the German Protection of Animals Act (TierSchG §4), i.e. animals were deeply anesthetized and euthanized with an overdose of MS222. According to this, no ethical approval is required.

### Gross inner ear morphology

#### Contrast enhanced microCT imaging

For a three-dimensional visualization of inner ears, contrast enhanced microCT scans of one specimen per species were performed (*E. maculatus*, SL = 33 mm; *S. tinanti*, SL = 39 mm; *H. guttatus*, SL = 39 mm). Animals were anesthetized and euthanized with an overdose (approx. 0.02%) of MS222 (Sigma-Aldrich, Vienna, Austria). Subsequently, the abdomen was ventrally opened to facilitate penetration of the fixative and staining solution. Fishes were then fixed in 10% buffered formalin (buffered in 0.1 M cacodylate buffer, pH = 7.2) for up to five days at 4°C, dehydrated in an ascending methanol series (50%, 75%, 100%; at least one hour each), and stained in 0.3% phosphotungstic acid in 100% methanol in order to enhance the tissue contrast (for details see [29]). The specimen of *S. tinanti* was reanalyzed from a previous study [29].

High-resolution microCT scans were performed with an Xradia MicroXCT high-resolution microCT system (Carl Zeiss X-Ray Microscopy, Pleasanton, CA) with a tungsten microfocus X-ray source and variable secondary optical magnification. The scans were made using an anode voltage of 40 kV at 4–8 W, an exposure time of 5–8 seconds, and 2 × 2 pixel binning for projection images every 0.25°. Each sample was scanned at two different magnifications, producing a final isotropic voxel size of 10 μm for illustration of both inner ears including the otoliths and an isotropic voxel size of 4 μm for visualizing the sensory epithelia (cristae and maculae) and otoliths of one inner ear per species.

#### Segmentation and 3D rendering

3D rendering of inner ear structures (including otoliths and sensory epithelia) was accomplished using AMIRA® v. 5.4.1 (Visage Imaging GmbH, Berlin, Germany). For labeling structures in the microCT generated stack, mainly a threshold-based segmentation was applied; if necessary, this labeling was refined or corrected using the brush tool. For the image stack based on the histological serial sectioning, manual labeling was performed by using the brush tool only. For reconstructing the macula lagenae of *E. maculatus*, initially every 3^rd^ image was labeled, with subsequent interpolation of structures on intervening images, followed by a manual check and correction of segmentation results if required (for further details see [29]).

Subsequently, every otolith and organ was separated from the ‘master’ LabelField file into single LabelFields and saved as separate files. Surface rendering was performed with the SurfaceGen module. If necessary, surfaces of each labeled object were reduced to 100,000 surfaces. This was followed by smoothing the surfaces using the SmoothSurface module (20 iterations; unconstrained smoothing).

In addition to the 3D reconstructions based on the microCT scans, the macula lagenae of *E. maculatus* was also reconstructed using a histological section series from a recent study [30]. For the histological image stack (final voxel size: x = y = 0.54 μm; z = 3 μm), manual labeling was performed by using the brush tool only. Initially every 3^rd^ image was labeled. Interpolation and creation of the surface was identical to the procedure described above.

#### Interactive 3D models

To facilitate an easy assessment of the reconstructed inner ears, including the maculae and their respective orientation patterns of ciliary bundles in 3D, we created interactive figures giving the viewer a free choice of perspective and organ composition. Interactive 3D pdf models were created using Adobe Acrobat 9 pro extended (Adobe Systems, San Jose, CA, USA) and Deep Exploration 5.5 (right hemisphere). We adopted the procedure described by Ruthensteiner and Heß [31], which was modified as follows: the 3D surface renderings of each organ system were exported as wavefront-files, simplified and colored in Deep Exploration and assembled to complex u3d-files, and finally embedded in PDF files with predefined views using the multimedia-tool of Acrobat 9 pro extended.

#### Additional dissections

In order to validate the outcomes of the 3D reconstructions of the sensory epithelia, we dissected the three specimens used for high-resolution microCT. Moreover, we additionally dissected samples fixed in 10% formalin which were partly used in a previous study of swim bladder morphology and hearing abilities [25] (six specimens of *E. maculatus*, SL: 25–41 mm; four specimens of *S. tinanti*, SL: 42–63 mm; three specimens of *H. guttatus*, SL: 45–55 mm) and dissected and stained (with 1% osmium tetroxide solution) one inner ear in two further specimens of *E. maculatus* (SL: 47, 49 mm) and one further individual of *S. tinanti* (SL: 56 mm) .

### Sensory epithelia

#### Immunostaining

To identify orientation patterns, ciliary bundles of the maculae were stained according to Lu and Popper [32] with TRITC-labeled phalloidin (Sigma-Aldrich, St. Louis, MO, U.S.A.) for stereocilia and anti-bovine α-tubulin mouse monoclonal antibodies (Molecular Probes®, Invitrogen, Darmstadt, Germany) and Alexa Fluor 488 conjugated anti-mouse secondary antibodies (Molecular Probes®, Eugene, OR, U.S.A.) for kinocilia. Prior to staining, heads were cut medio-sagittally and fixed for 1–2 hours in 10% buffered (0.1 M phosphate buffer) formalin solution at room temperature. Inner ears were dissected out in fixative, otoliths removed, after which maculae were washed four times at room temperature in 0.1 M phosphate buffer at 20 minute intervals on a slowly moving shaker. All further steps (unless specified otherwise) were performed at room temperature on a shaker, and after every staining/antibody exposure step tissue samples were washed four times with phosphate buffer with sodium azide at 20 minute intervals. Inner ears were incubated in blocking solution for 1 h and then incubated overnight in anti-bovine α-tubulin mouse monoclonal antibodies (1:200 dilution in 0.1 M phosphate buffer with sodium azide). Sensory epithelia were incubated in Alexa Fluor 488 anti-mouse antibodies (1:200 dilution in 0.1 M phosphate buffer) for 1.5 h at 37°C and in TRITC-labeled phalloidin (1:100 dilution in 0.1 M phosphate buffer) for 4 to 5 h. After the staining procedure, samples were stored at 4°C for one day, after which the sensory epithelia were mounted on a slide with an anti-fading medium, VectaShield® (Vector Laboratories). In this medium, the maculae were carefully flattened and then covered with a cover slip, sealed with nail polish, and stored at 4°C.

#### Confocal imaging

Samples were investigated with a Leica TCS SP2 inverse confocal laser scanning microscope (CLSM) using a Leica HCX PL APO UV 40× oil immersion objective (NA = 1.25) and with the 488 nm Ar and the 543 nm He-Ne laser lines. Staining with TRITC-labeled phalloidin clearly showed the labeled stereocilia and a ‘hole’ for the non-labeled kinocilium, while kinocilium-staining (Alexa Flour 488) displayed the specific labeling of kinocilia only.

#### Quantification of the number of ciliary bundles and the macula area

**Figure 1:**
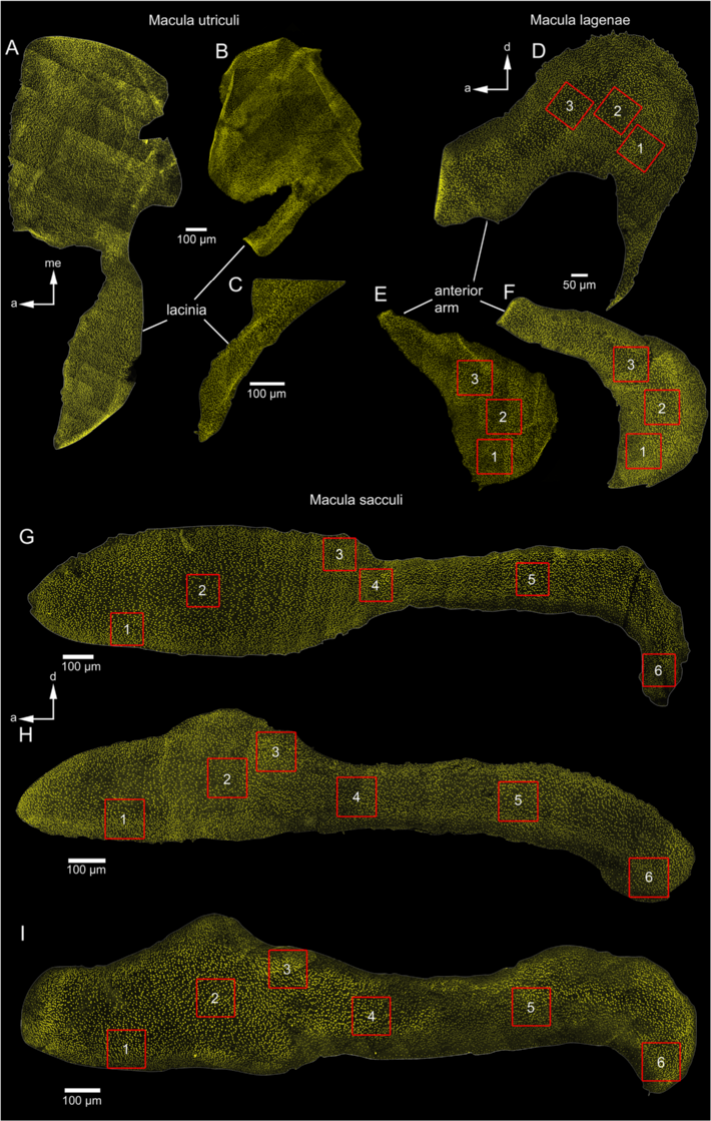
**Phalloidin-labeled macula utriculi, macula sacculi and macula lagenae of the three investigated cichlid species. ***E. maculatus* shows a distinctly broader lacinia of the macula utriculi **(A)**, a larger macula lagenae **(D)**, and a wider ostium (see explanation in Figure 5) of the macula sacculi **(G)** than *H. guttatus***(C, F, I)** and *S. tinanti***(B, E, H)**. The red squares indicate the patches on the macula lagenae and the macula sacculi chosen for ciliary bundle counts. a, anterior; d, dorsal; me, medial. Scale bars, 100 μm **(A-C, G-I)**, 50 μm **(D-F)**.

For the analysis of the maculae overlapping image stacks were photographed at a z-step size of 1 μm and a pixel size of 183 nm × 183 nm (e.g., up to 50 stacks for the macula sacculi and the macula utriculi). The image stacks were reduced to one image each by applying the brightest point projection tool in Image J v. 1.46r. Brightest point projected images were used for creating coherent maps of the stained stereocilia of each macula by manual stitching in Adobe Photoshop CS4®. Orientation patterns of ciliary bundles were determined as described by Lu and Popper [32] and densities of ciliary bundles on the maculae evaluated on squares (in the following termed “patches”) comprising an area of 10,000 μm^2^ each (Figure 1). Ciliary bundles were counted using Image J v. 1.46r (see also [33]), counts were additionally checked on printouts and—if necessary—measures were corrected accordingly. The total number of ciliary bundles on the macula sacculi (Hms) was estimated using the equation (see [34]):

$$\begin{array}{ll} \;\text{Hms} & =[\left(\text{sum}\;\text{over}\;\text{patch}\;1\;\text{to}\;3\right)\\ & *\left(\text{area}\;\text{of}\;\text{the}\;\text{ostium}/\text{area}\;\text{of}\;\text{all}\;\text{patches}\;\text{in}\;\text{the}\;\text{ostium}\right)]\\ & \;+[\left(\text{sum}\;\text{over}\;\text{patch}\;1\;\text{to}\;3\right)\\ & *\left(\text{area}\;\text{of}\;\text{the}\;\text{cauda}/\text{area}\;\text{of}\;\text{all}\;\text{patches}\;\text{in}\;\text{the}\;\text{cauda}\right)] \end{array}$$

Accordingly, the total number of ciliary bundles on the macula lagenae (Hml) was estimated as follows:

$$\begin{array}{ll} \;\text{Hml}= & \left(\text{sum}\;\text{over}\;\text{patch}\;1\;\text{to}\;3\right)\\ & *\left(\text{macula}\;\text{area}/\text{area}\;\text{of}\;\text{all}\;\text{patches}\right). \end{array}$$

The surface area of the maculae sacculi and maculae lagenae was determined as follows: The outlines of the maps generated from the stained stereocilia were drawn in Adobe Illustrator CS4®. Then a bmp file was generated in Adobe Photoshop CS4® showing a white macula on a black background. These bmp files were opened in tpsDig2 [35], and the area of each macula was determined applying the ‘ImageTools–Measure’ option considering proper scales.

#### Ciliary bundle types

Investigation of ciliary bundle types was performed in *E. maculatus* and *S. tinanti*. Heads were cut medio-sagittally and fixed in 2% glutaraldehyde solution in 0.1 M phosphate buffer overnight at 4°C. Inner ears were then dissected and washed three times with 0.1 M cacodylate buffer. Subsequently, otoliths were dissected and the otolithic membrane was removed. Sensory epithelia were postfixed with 1% osmium tetroxide solution in 0.1 M cacodylate buffer on ice for 1–2 h. Then sensory epithelia were dehydrated through a graded acetone series (30%, 50%, 70%, 80%, 90%, followed by 3 steps with 100% acetone) at 20 minutes intervals prior to critical point drying, for which carbon dioxide was used as intermediary fluid using a Leica EM CPD300. The maculae were mounted on aluminum stubs medial-side up (macula sacculi and macula lagenae) or dorsal-side up (macula utriculi) with platelets of conductive glue or on conductive plasticine (Leit-C-Plast, Plano GmbH). The stubs were coated with gold using an AGAR B7340 sputter coater, and images were taken using a Philips XL 20 SEM at 15 kV. Ciliary bundle types were identified on the resulting SEM images at magnifications of 3,000- to 15,000.

#### Projection of 2D orientation patterns on 3D models of the maculae

In order to illustrate 2D orientation patterns of ciliary bundles on the 3D models of the maculae and thus visualizing them in their natural 3D curvature we applied two different methods. (1) We projected 2D drawings of the orientation patterns of ciliary bundles on the respective 3D models of the maculae (macula lagenae) using Deep Exploration 5.5 (right hemisphere). (2) surfaces of the maculae created and rendered in AMIRA® v. 5.4.1 were opened in Adobe Photoshop CS6® Extended as wavefront (obj) files. Then arrows indicating the orientation groups of ciliary bundles were drawn on the dorsal face of the 3D models of the macula utriculi using the 3D tools in Photoshop that also allowed taking snapshots of the resulting 3D orientation patterns.

For creation of an interactive model of 3D orientation patterns, the 3D model and the drawn arrows were exported as separate files (3D model: wavefront file, drawing: tif file). Subsequently, the files were both opened in Deep Exploration 5.5 (right hemisphere) in which arrows (tif file) were projected on the 3D model and exported as one u3d file that could be used for an interactively accessed pdf (see ‘Interactive 3D models’).

### Otoliths

Otoliths removed from the sensory epithelia used for immunostaining were mechanically cleaned of organic remains in distilled water using a pair of fine tweezers and then stored dry in plastic boxes. Otoliths were not affected by the short fixation period (1–2 h) in the buffered formalin solution (e.g., [36]).

In order to evaluate otolith surface area either left or right saccular and lagenar otoliths from each specimen were positioned plainly, with their medial face upward-oriented and were inspected under a stereomicroscope (Leica MZ 6; camera: Leica DFC 295). The surface area of the utricular otoliths was not quantified because this otolith type was strongly convex in *E. maculatus* while rather flat in *S. tinanti* and *H. guttatus*, which would have resulted in a substantial underestimation of otolith area in the former species. Digital images were taken at maximum magnifications using the Leica ImageAccess Standard 8 (Imagic AG, Glattbrugg, Switzerland) applying the multifocus option (extended focus imaging). All digital images were processed in Adobe Photoshop CS4® by generating a contrast of 100% along the contour. To determine otolith area, the orthogonal projection of the area of the macula-oriented face of each otolith type was measured using tpsDig 2 [35]. Otolith weight was determined to the nearest 0.01 mg with a Mettler-Toledo® AT21 (measurement error: ± 0.012 mg).

To illustrate otolith morphology, two representative otolith triplets, i.e. utricular, saccular, and lagenar otoliths per species were mounted on aluminum stubs with platelets of conductive glue or plasticine (Leit-C-Plast, Plano GmbH), and coated with gold using a Polaron E 5100 sputter coater. Images were taken with a LEO 1430VP SEM at 13 kV.

### Statistical analyses

All statistical analyses were conducted in PASW Statistics 18 (SPSS Inc.). All variables were ln-transformed to account for non-linear relationships between variables. General Linear Models (GLM) fulfilled the criterion of homogeneity of variances (Levene’s tests: *P* > 0.05). We calculated the following univariate GLMs using: (1) macula surface area as dependent variable, ‘species’ as a fixed factor, and ‘standard length’ as a covariate; (2) numbers of ciliary bundles as the dependent variable, ‘species’ as a fixed factor, and ‘macula area’ as covariate; (3) otolith area as dependent variable, ‘species’ as fixed factor, and ‘standard length’ as a covariate; (4) otolith weight as the dependent variable while using ‘species’ as a fixed factor, and ‘body weight’ as a covariate. We initially included interaction terms between the fixed factors and covariates, but as they were not significant (*P* > 0.05), final models contained main effects only.

To analyze potential differences in head height of species—which may have an effect on inner ear morphology—we ln-transformed head height (HH) and standard length (SL) and treated lnHH as the dependent variable, lnSL as a covariate, and ‘species’ as a fixed factor in an ANCOVA; again, the interaction term was excluded as it was not significant (*P* > 0.05).

## Results

### Differences in gross inner ear morphology

**Figure 2:**
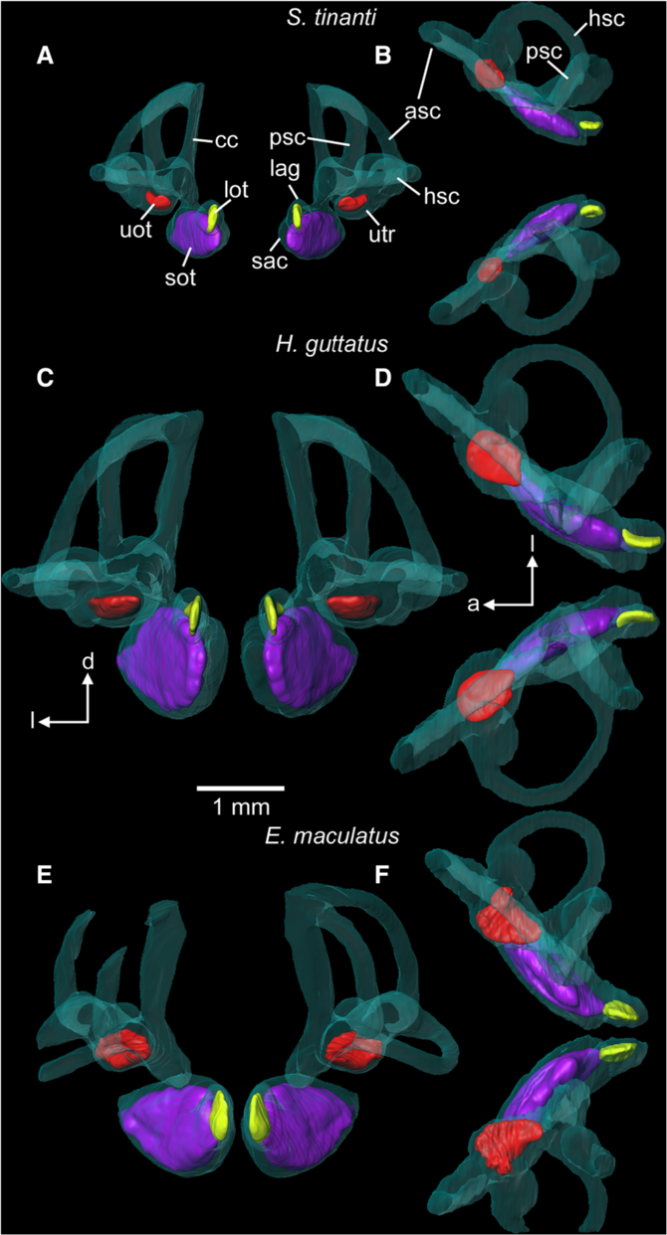
**Overview of left and right inner ears including otoliths in (A-B) *S. tinanti*, (C-D) *H. guttatus*, and (E-F) *E. maculatus*.** Inner ears are shown in posterior **(A, C, E)** and dorsal views **(B, D, F)**. While inner ears are rather close to each other in *H. guttatus* and *E. maculatus*, the distance between left and right ears is distinctly larger in *S. tinanti*. In *E. maculatus* the lagenae come very close to each other and the spatial orientation of the semicircular canals—especially of the horizontal semicircular canal—differ from the other two species. Note that semicircular canals of the left inner ear of *E. maculatus* are incomplete. The 3D reconstructions are based on microCT scans (isotropic voxel size: 10 μm). a, anterior; asc, anterior semicircular canal; cc, common canal; d, dorsal; hsc, horizontal semicircular canal; l, lateral; lag, lagena; lot, lagenar otolith; psc, posterior semicircular canal; sac, saccule; sot, saccular otolith; uot, utricular otolith; utr, utricle. Scale bar, 1 mm.

**Figure 3:**
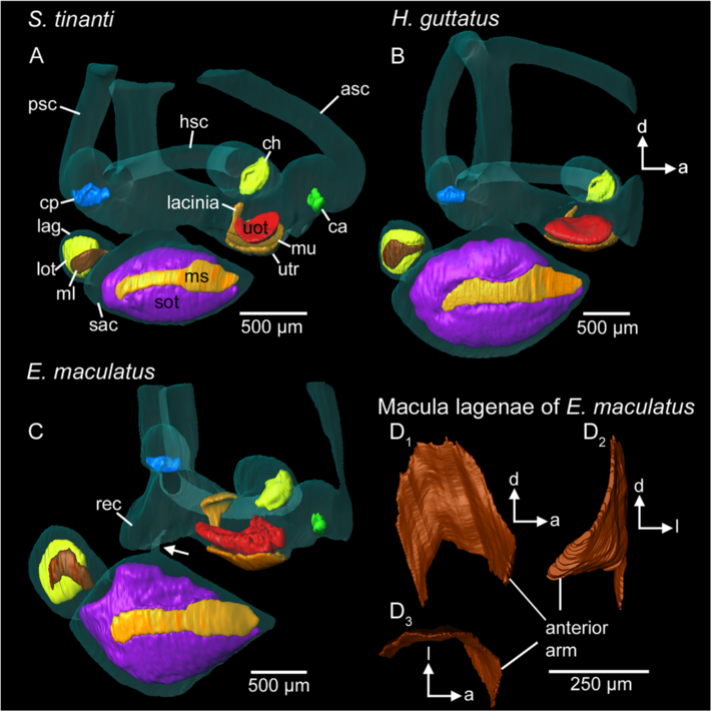
**Spatial orientation and curvature of sensory epithelia (maculae and cristae) in the studied species.** While the anterior arm of the macula lagenae is directed upward in *S. tinanti***(A)** and *H. guttatus***(B)**, it points anteriorly in *E. maculatus***(C, D)**. A 3D model of the macula lagenae of *E. maculatus* based on histological serial sections illustrates the strong curvature of the anterior arm **(D**_**1**_**-D**_**3**_**)**. In *E. maculatus***(C)**, part of the lacinia of the macula utriculi forms a dorsal “roof” above the utricular otolith. The white arrow in **(C)** marks the thin connection between saccule and the upper part of the inner ear. 3D reconstructions are based on microCT scans (**A-C**, isotropic voxel size 4 μm) and histological section series (**D**, voxel size x = y = 0.54 μm; z = 3 μm). a, anterior; ca, crista of the anterior semicircular canal; ch, crista of the horizontal semicircular canal; cp, crista of the posterior semicircular canal; d, dorsal; l, lateral; lag, lagena; lot, lagenar otolith; ml, macula lagenae; ms, macula sacculi; mu, macula utriculi; rec, recessus situated posteriorly to the utricle; sac, saccule; sot, saccular otolith; uot, utricular otolith; utr, utricle. Scale bars, 500 μm **(A-C)**, 250 μm **(D)**.

We observed distinct differences between species in overall shape of the inner ears (Figure 2). The head in *S. tinanti* was significantly flatter than in *E. maculatus* and *H. guttatus* (ANCOVA, *F*_2, 19_ = 505.2, *P* < 0.001; Table 1). Accordingly, the inner ear was dorso-ventrally compressed in *S. tinanti*, showing shorter vertical semicircular canals than *E. maculatus* and *H. guttatus* (Figure 3A *vs.* B-C). The saccule was elongate and oval in *S. tinanti* (Figure 3A), while it was taller and oval in *H. guttatus*, and possessed a pointed tip in *E. maculatus* (Figure 3B-C). Inner ears of *E. maculatus* displayed a distinctly thinner connection between the saccule, the semicircular canals and the utricle (Figure 3 C *vs.* A-B) than those of *H. guttatus* and *S. tinanti*. Moreover, left and right lagenae were situated distinctly closer to each other in *E. maculatus* (Figure 2E-F). While the horizontal semicircular canal was oriented exactly along the horizontal plane in *S. tinanti* and *H. guttatus* (Figure 2A, C), it was slightly ventrally bent in *E. maculatus* (Figure 2E), resulting in an upward shift of the respective crista (Figure 3C).

**Table 1:** **Overview of samples sizes (*N*) of the three cichlid species and mean values (±s.e.m.) of standard length (SL), head length (HL), head height (HH), body weight (BW), numbers of maculae and otolith triplets analyzed**

**Species**	***N *** **(f/m/im)**	**SL [mm]**	**HL [mm]**	**HH [mm]**	**BW [g]**	***N *** **maculae (mu/ms/ml)**	***N *** **otolith triplets**
***S. tinanti***	8 (3/3/2)	57 ± 4.9	16 ± 1.6	12 ± 1.2	3.2 ± 0.85	3/6/4	8
***H. guttatus***	5 (3/1/1)	40 ± 0.5	14 ± 0.2	14 ± 0.2	2.1 ± 0.09	3/5/4	5
***E. maculatus***	8 (3/4/1)	47 ± 1.6	18 ± 0.7	21 ± 0.9	4.2 ± 0.48	3/5/7	7

f, female; im, immature; m, male; ml, macula lagenae; ms, macula sacculi; mu, macula utriculi.

### Maculae

**Figure 4:**
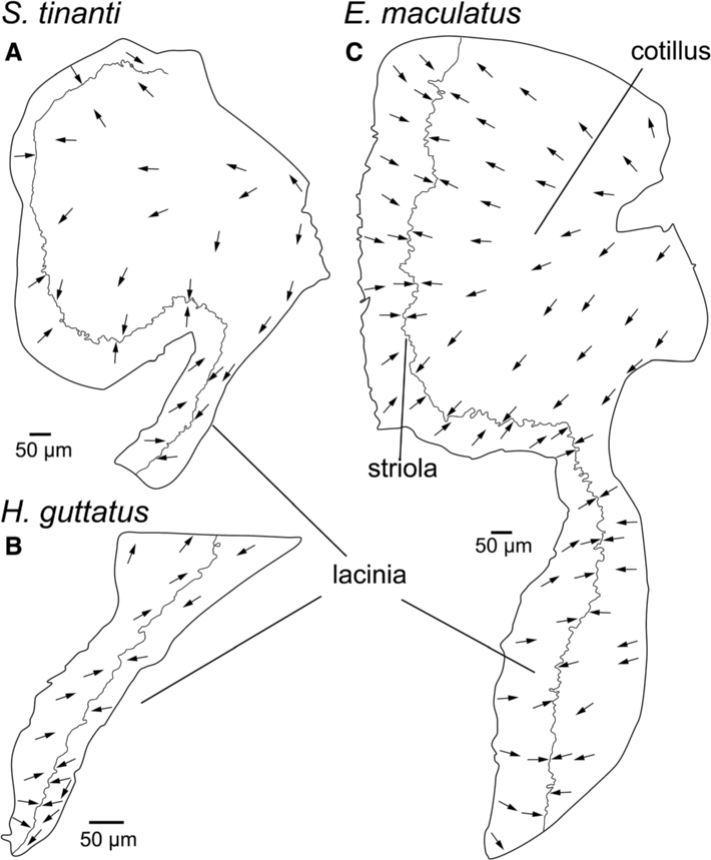
**Interspecific comparison of the orientation patterns of ciliary bundles on the macula utriculi.** The orientation pattern in the cotillus and striola regions of the macula utriculi is similar in all three species while ciliary bundles on the lacinia of *E. maculatus***(C)** show a more opposing orientation than those on the lacinia of *S. tinanti***(A)** and *H. guttatus***(B)**. Note that the arrows point into the direction of the kinocilia indicating the orientation of the ciliary bundles in the respective area while the dashed lines separate different orientation groups. Scale bars, 50 μm.

In all three species, the macula utriculi was bowl-shaped and showed a lateral extension, the lacinia (compare Additional files 1, 2 and 3). In *E. maculatus* the lacinia was distinctly larger and extended from lateral to dorsal, forming a roof-like structure above the utricular otolith (Figure 3C). The orientation pattern of ciliary bundles on the macula utriculi displayed a radial arrangement of ciliary bundles on the cotillus, and an opposing pattern in the striola region (Figure 4A-C); on the lacinia the ciliary bundles were opposing in *E. maculatus* (Figure 4C), but rather antiparallel in *S. tinanti* (Figure 4A) and *H. guttatus* (Figure 4B).

**Figure 5:**
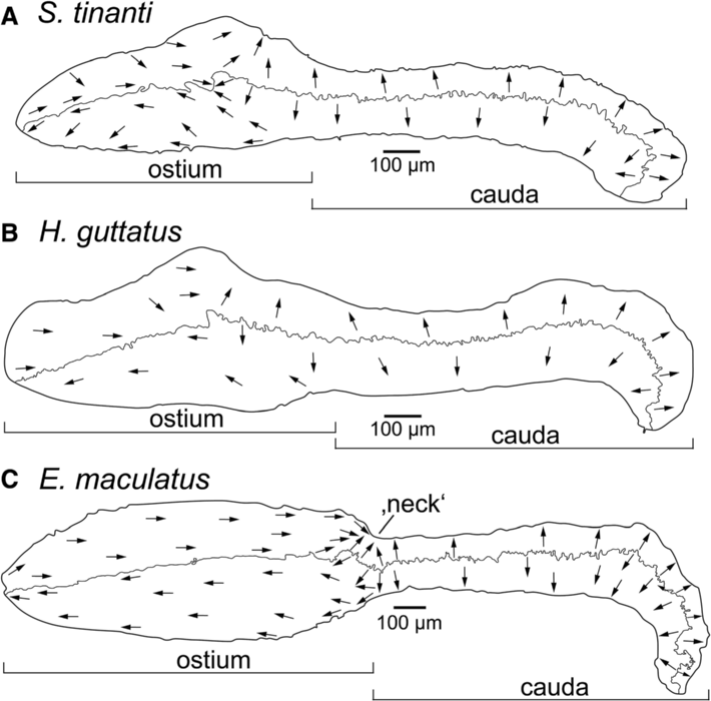
**Inter-specific comparison of the orientation patterns of ciliary bundles on the macula sacculi.** Ciliary bundles of all three species **(A-C)** are arranged into two horizontal groups on the ostium, two vertical groups on the cauda, and again two horizontal groups in the posteriormost region of the cauda of the macula. Note that the arrows point into the direction of the kinocilia indicating the orientation of the ciliary bundles in the respective area while the dashed lines separate different orientation groups. Scale bars, 100 μm.

The macula sacculi of *E. maculatus* showed a larger ostium (Table 2) that was distinctly separated from the cauda by a “neck” while the macula sacculi of the other two species showed a gradual transition from the ostial to the caudal region (Figure 1). The posterior tip of the cauda in *E. maculatus* was more distinctly bent in ventral direction. ‘SL’ and ‘species’ had a significant effect on macula area (Table 3) with *E. maculatus* showing slightly larger maculae sacculi than *H. guttatus* and distinctly larger ones than *S. tinanti* (Table 2). The orientation pattern of ciliary bundles on the macula sacculi was the same for all three species (Figure 5A-C) and resembled the “dual” pattern described by Popper and Coombs [18]; two horizontally oriented groups on the anterior part of the ostium, two vertical groups on the posterior part of the ostium and large parts of the cauda, and again two horizontal groups on the posterior-most portion of the cauda. Though density of ciliary bundles at the anterior, dorsal and posterior margins was higher than in the central portion of the macula sacculi, all three species showed a distinctly lower density on the ventral margin of the posterior part of the ostium and along the whole ventral margin of the cauda (Figure 1). The estimated total number of ciliary bundles was significantly affected by ‘macula area’ and also differed between species (Table 2). *E. maculatus* and *H. guttatus* displayed more ciliary bundles than *S. tinanti* (Tables 2 and 3).

**Table 2:** Mean values (±s.e.m.) of the area, number of ciliary bundles (no. of cbs) and estimated total number of ciliary bundles of the macula sacculi and macula lagenae, respectively, as well as mean values (±s.e.m.) for area and weight of otoliths and three ratios discussed in the main text

			***S. tinanti***	***H. guttatus***	***E. maculatus***
**Macula sacculi (Ms)**	Area [μm^2^]		306,620 ± 48,634	522,290 ± 35,764	619,646 ± 24,902
	No. of cbs	1	175 ± 12.8	139 ± 9.0	130 ± 5.6
		2	263 ± 5.4	274 ± 13.6	209 ± 8.4
		3	271 ± 4.7	243 ± 19.4	319 ± 7.5
		4	306 ± 21.7	209 ± 17.9	289 ± 6.6
		5	245 ± 13.3	178 ± 12.3	220 ± 4.8
		6	254 ± 12.8	300 ± 13.1	326 ± 7.1
	Estimated total no.		7,734 ± 1,078	11,430 ± 575	14,871 ± 725
**Macula lagenae (Ml)**	Area [μm^2^]		86,671 ± 10,484	131,282 ± 4,537	307,651 ± 16,802
	No. of cbs	1	506 ± 7.5	424 ± 17.6	321 ± 10.5
		2	345 ± 12.4	295 ± 10.3	248 ± 21.6
		3	370 ± 5.3	312 ± 9.8	260 ± 14.8
	Estimated total no.		3,491 ± 755	4,494 ± 127	8,297 ± 646
**Saccular otolith (SO)**	Area [μm^2^]		907,214 ± 138,269	1,856,923 ± 34,540	2,301,892 ± 90,444
	Weight [μg]		598 ± 116	1,319 ± 29	2,039 ± 125
**Lagenar otolith (LO)**	Area [μm^2^]		108,403 ± 9,991	212,385 ± 6,912	391,103 ± 14,588
	Weight [μg]		34 ± 1.7	145 ± 4.5	83 ± 4.1
**Utricular otolith (UO)**	Weight [μg]		92 ± 8.3	37 ± 3.5	220 ± 11.3
**Ratios**	Area (Ms/Ml)		3.4 ± 0.24	4.0 ± 0.37	2.0 ± 0.05
	Area (Ostium/Cauda)		0.9 ± 0.04	1.0 ± 0.05	1.7 ± 0.05
	Area (SO/LO)		8.1 ± 0.54	8.8 ± 0.32	5.9 ± 0.16

**Table 3:** Full factorial general linear models (GLM) using the ln-transformed area or number of ciliary bundles (no. of cbs) of the respective macula as dependent variable and the respective ln-transformed standard length (SL) or macula area (area) as covariate

**Source**		***df***	**Mean square**	***F***	***P***	**η**^**2**^
**Macula sacculi**						
**Ln(area)**	Species	2	1.496	162.91	**<0.001**	0.96
	Ln(SL)	1	1.015	110.54	**<0.001**	0.90
	Error	13	0.009			
**Ln(no. of cbs)**	Species	2	0.017	4.15	**0.040**	0.39
	Ln(area)	1	0.789	188.56	**<0.001**	0.94
	Error	13	0.004			
**Macula lagenae**						
**Ln(area)**	Species	2	3.028	261.72	**<0.001**	0.97
	Ln(SL)	1	0.0410	35.48	**<0.001**	0.72
	Error	14	0.012			
**Ln(no. of cbs)**	Species	2	0.037	4.69	**0.045**	0.54
	Ln(area)	1	0.647	82.90	**<0.001**	0.91
	Error	8	0.008			

Significant *P*-values are given in bold.

**Figure 6:**
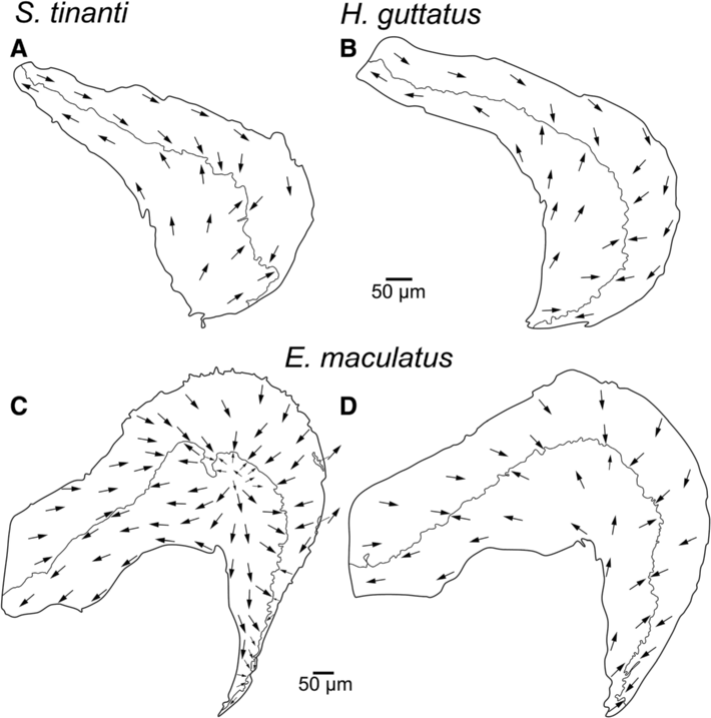
**Interspecific comparison of the orientation patterns of ciliary bundles on the macula lagenae.** Ciliary bundles of all species are mainly arranged into two “opposing” groups **(A-B, D)**. In *E. maculatus*, however, distinct intraspecific variability can be observed as shown in **(C)***vs.***(D)**. In **(C)**, ciliary bundles in the center of the macula show a radial arrangement. Note that the arrows point into the direction of the kinocilia indicating the orientation of the ciliary bundles in the respective area while the dashed lines separate different orientation groups. Scale bars, 50 μm.

The macula lagenae in *E. maculatus* displayed a large anterior arm that was bent anteriorly while the anterior arm was much smaller and antero-dorsally oriented in *H. guttatus* and *S. tinanti* (Figure 1D *vs.* E-F; Figure 3C *vs.* A-B; D). ‘Standard length’ and ‘species’ significantly affected macula area, with *E. maculatus* possessing a distinctly lager macula lagenae than both other species (Tables 2 and 3). Two major orientation groups of ciliary bundles were found on the macula lagenae in all three species, and ciliary bundles showed a slightly opposing to antiparallel pattern (Figure 6A-B, D). While the orientation pattern was consistent in all studied specimens of *H. guttatus* and *S. tinanti*, five out of seven maculae of *E. maculatus* showed deviations of this pattern in that they showed small patches of additional orientation groups at the posterior margin (five out of seven maculae) and a radial (two maculae) (Figure 6C) or a whirl-like arrangement of ciliary bundles (one macula) in the center. The estimated total number of ciliary bundles was a function of ‘macula area’ and differed significantly between species, with *E. maculatus* possessing distinctly more ciliary bundles compared to *H. guttatus* and *S. tinanti* (Tables 2 and 3). No macula neglecta was found in any of the three species.

The ostium of the macula sacculi in *E. maculatus* was distinctly larger than the cauda; in *S. tinanti* and *H. guttatus* ostium and cauda were of equal size (Table 2). The macula sacculi in *E. maculatus* was twice as large as the macula lagenae, while it was three to four times larger in *S. tinanti* and *H. guttatus* (Table 2).

**Figure 7:**
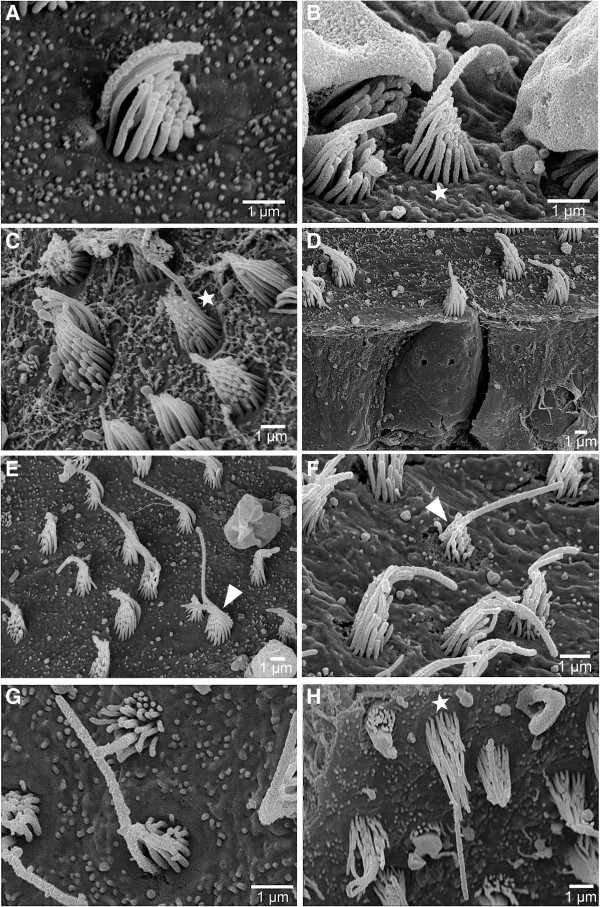
**Overview of ciliary bundle types.** Ciliary bundle types identified on the maculae of *E. maculatus***(A, C, D, E)** were similar to those found on the maculae of *S. tinanti***(B, F, G, H)**. Both species showed ciliary bundles in which the kinocilium was only slightly longer than stereocilia **(A, D)**, approximately twice as long as the longest stereocilium (**B, C, H,** marked with an asterisk), or ciliary bundles with a kinocilium at least three times longer than the longest stereocilium (**E-G** marked with an arrowhead). Scale bars, 1 μm.

*E. maculatus* and *S. tinanti* showed no obvious differences of ciliary bundle types on any of the maculae. The cotillus of the macula utriculi was mainly characterized by short ciliary bundles in which the kinocilium was either slightly longer than or twice as long as the longest stereocilium while in the striola region and partly on the lacinia ciliary bundles were taller. On the macula sacculi and macula lagenae, ciliary bundles were short, with kinocilia being slightly longer than (Figure 7A, D), or twice as long as the longest stereocilium (Figure 7B-C, H) in the central portion of the maculae. Ciliary bundles had long kinocilia on the margins (Figure 7E-G).

### Orientation patterns of ciliary bundles in 3D

While our 2D analysis of orientation patterns of ciliary bundles on the three macula types showed rather similar patterns, inter-specific differences become apparent when illustrating the orientation patterns on the 3D models of the maculae (Additional file 4). Beside the cotillus, the macula utriculi in *E. maculatus* shows an additional horizontal plane on which ciliary bundles are arranged on the roof-like portion of the lacinia (Additional file 4C). An arrangement approximately along the transversal axis could be observed on the anterior arm of the macula lagenae (Figure 3D). While the macula lagenae was uniformly vertically oriented in *S. tinanti* and *H. guttatus*, it was bent up to 90° in the anterior-most portion in *E. maculatus* (Figure 3D_3_), resulting in a two-planar sensory epithelium and thus, additional orientation groups of ciliary bundles on this macula (Additional file 4D).

### Otoliths

**Figure 8:**
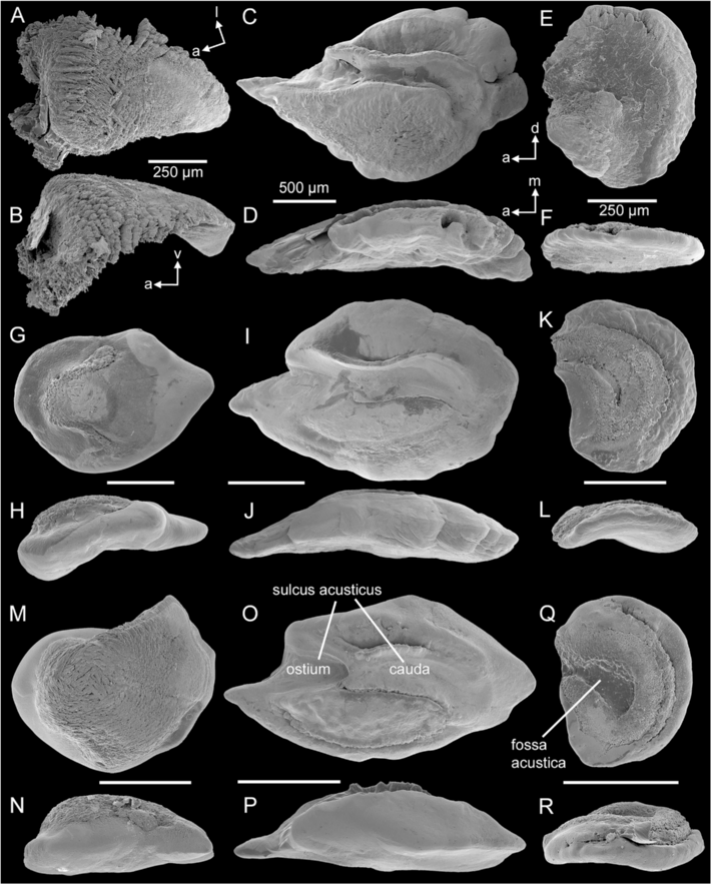
**SEM micrographs of the three otolith types in the studied cichlid species.** Utricular, saccular, and lagenar otoliths of *E. maculatus***(A-F)**, *H. guttatus***(G-L)**, and *S. tinanti***(M-R)** are shown in ventral **(A, G, M)** or medial views **(C, E, I, K, O, Q)**, and in lateral **(B, H, N)** or dorsal views **(D, F, J, L, P, R)**. Scale bars, 250 μm (utricular and lagenar otoliths), 500 μm (saccular otoliths). a, anterior; d, dorsal; l, lateral; m, medial; v, ventral.

In ventral view, the utricular otolith was slightly curved in *E. maculatus* while it was diamond-shaped in *S. tinanti* and *H. guttatus* (Figure 8A *vs.* G, M). In lateral view, the utricular otolith was strongly convex in *E. maculatus* whereas it was rather flat in the other species (Figure 8B *vs.* H, N). In contrast to the smooth surface of the utricular otoliths of *H. guttatus* and *S. tinanti*, those of *E. maculatus* were characterized by conspicuously elongated crystal regions partly forming platelet- or needle-like structures. In freshly dissected otoliths of *E. maculatus*, the space between these crystal regions was filled with otolithic membrane on the ventral and the dorsal faces.

The saccular otolith was more rhombic with a distinctly pointed anterior tip (rostrum tip) in *E. maculatus* (Figure 8C), oval shaped in *H. guttatus* (Figure 8I), and elongate oval in *S. tinanti* (Figure 8O). In dorsal view, saccular otoliths of all three species were moderately to distinctly thickened and convex (Figure 8D, J, P). In all three species the furrow housing the macula sacculi, namely the sulcus acusticus was heterosulcoid, i.e. divided into an ostial (anterior) and a caudal (posterior) part, and opened at its anterior and closed at its posterior ends (Figure 8O).

The lagenar otolith in *E. maculatus* was extremely thin, flat (Figure 8F), and disc-shaped (Figure 8E) and the furrow housing the macula lagenae (fossa acustica) was boomerang-shaped with its open end pointing anteriorly; in lagenar otoliths of *H. guttatus* and *S. tinanti*, this furrow was only slightly curving and its open end pointed antero-dorsally (Figure 8K, Q). Moreover, otoliths in these two species were oval shaped and slightly thickened along the medio-lateral axis (Figure 8K-L, Q-R).

The species differed significantly with respect to the area of the saccular and lagenar otoliths and the weight of all three otolith types (Table 4). Saccular and lagenar otoliths showed a smaller area and lower weight in *S. tinanti* compared to the other species (Table 2). In contrast to the saccular otolith, the lagenar otolith of *E. maculatus* possessed a distinctly lower weight than that in *H. guttatus* (Table 2). The utricular otolith was heaviest in *E. maculatus* and lightest in *H. guttatus* (Table 2). Similar to the ratio of the area of macula sacculi to that of the macula lagenae (see above), lagenar otoliths were distinctly smaller than the saccular otolith in *S. tinanti* and *H. guttatus* whereas this size difference between the two otolith types was less pronounced in *E. maculatus* (Table 2).

**Table 4:** Full factorial GLMs using the ln-transformed area or weight of the respective otolith type as dependent variable and ln-transformed standard length (SL) or body weight (BW) as covariate

**Source**		***df***	**Mean square**	***F***	***P***	**η**^**2**^
**Saccular otolith**	**Ln(area)**					
	Species	2	2.719	235.945	**<0.001**	0.967
	Ln(SL)	1	1.267	109.925	**<0.001**	0.873
	Error	16	0.012			
	**Ln(weight)**					
	Species	2	2.735	120.753	**<0.001**	0.938
	Ln(BW)	1	2.153	95.054	**<0.001**	0.856
	Error	16	0.023			
**Lagenar otolith**	**Ln(area)**					
	Species	2	3.288	480.964	**<0.001**	0.984
	Ln(SL)	1	0.426	62.392	**<0.001**	0.796
	Error	16	0.007			
	**Ln(weight)**					
	Species	2	3.431	253.308	**<0.001**	0.969
	Ln(BW)	1	0.035	2.605	0.126	0.140
	Error	16	0.014			
**Utricular otolith**	**Ln(weight)**					
	Species	2	2.468	82.181	**<0.001**	0.922
	Ln(BW)	1	0.176	5.846	**0.030**	0.295
	Error	14	0.030			

Significant *P*-values are given in bold.

The antero-dorsal most part of the macula sacculi, the anterior most region of the anterior arm of the macula lagenae, and the lacinia of the macula utriculi were covered by otolithic membrane only.

## Discussion

We investigated whether modifications of the swim bladder in cichlid fishes, such as anterior extensions to the inner ears or a reduction of the entire swim bladder, are also reflected in modifications of inner ear structures, especially with respect to the sensory epithelia (maculae) of the otolithic end organs. We demonstrate that *E. maculatus*, a species with ancillary auditory structures (anterior swim bladder extensions) and enhanced auditory capacities (i.e., higher auditory sensitivities; [25,26]) also displays several modifications regarding inner ear morphology: (1) Certain portions of the maculae (macula utriculi: mainly the lacinia; macula sacculi: ostium; macula lagenae: anterior arm) were enlarged. (2) We found differences in the shape and curvature of the lacinia of the macula utriculi and the anterior arm of the macula lagenae that resulted in an altered arrangement of ciliary bundles when considering the natural orientation patterns in 3D. (3) The surface area of saccular and lagenar otoliths and the weight of saccular and utricular otoliths were increased. In contrast, inner ear morphology of *S. tinanti*, which has a vestigial swim bladder and poor auditory abilities, was similar to that seen in *H. guttatus*, which possesses a large swim bladder. Slight modifications of the inner ear in *S. tinanti* were apparent in the form of compressed semicircular canals, decreased areas and weights of saccular and lagenar otoliths, smaller areas of the macula sacculi and macula lagenae, and fewer ciliary bundles being found on the macula sacculi and macula lagenae. In *S. tinanti* these modifications may be explained primarily by the rheophilic lifestyle [37] and thus dorso-ventrally compressed body and head, leading to a compressed inner ear due to the limited space along the dorso-ventral axis.

### Similar hair cell orientation patterns in 2D, but interspecific differences in 3D

Artificially flattened maculae of *E. maculatus* exhibited an orientation pattern of hair cells that was similar in complexity to those in the other two cichlid species studied here as well as in several other teleosts without swim bladder extensions [38]. For example, similar orientation patterns of ciliary bundles on the macula sacculi have been described for the cichlids *Sarotherodon melanotheron* and *Andinoacara pulcher*[19] and other members of the order Perciformes (e.g., *Bathygobius fuscus*, *Gnatholepis anjerensis*[39]). Our findings are largely congruent with the results from a study on ciliary bundle orientation patterns in sciaenids with swim bladder extensions, which found similar orientation patterns in species with and without anterior swim bladder extensions [22] with the exception of the silver perch (*Bairdiella chrysoura*[23]). This finding demonstrates that there is not necessarily a direct relationship between ancillary auditory structures (anterior swim bladder extensions), enhanced auditory abilities, and more “complex” orientation patterns of ciliary bundles—at least when analyzing orientation patterns on artificially flattened maculae (i.e., in 2D).

In *E. maculatus* the most intimate contact exists between the swim bladder extensions and the lagenae, which could explain the unchanged orientation pattern of ciliary bundles on the macula sacculi. If this assumption were correct, one would still predict an altered orientation pattern and additional orientation groups on the macula lagenae, but again our analysis of artificially flattened maculae did not support this view. When considering the natural 3D curvature of the maculae, however, orientation patterns of ciliary bundles on the macula lagenae in *E. maculatus* did show a more complex pattern, as the ciliary bundles on the anterior-most portion of the macula lagenae formed additional orientation groups. Such a 3D arrangement of ciliary bundles was previously found in the anterior region (ostium) of the macula sacculi of silver perch [23], the ostium of the macula sacculi of the non-teleost bowfin *Amia calva* (Neopterygii, Amiidae) [40], and in the macula lagenae of the deep-sea dwelling elopomorph *Polyacanthonotus challenger*[41]. The wider range of directions of ciliary bundles based on the 3D curvature of the respective macula ought to translate into a wider range of directional stimuli being detectible, and hence ought to play a crucial role in sound source localization.

The altered morphology of the utricle (large and 3D-arranged lacinia of the macula utriculi and heavier utricular otoliths) in *E. maculatus* may be explained by two mutually non-exclusive hypotheses. First, the specialized utricle in *E. maculatus* may indicate an improvement of gravity perception and of linear acceleration perception, as the utricle is thought to be involved in the sense of balance (e.g., [42,43]). Second, the utricle in *E. maculatus* may also play a role in enhanced auditory abilities. For example, Popper and Tavolga [44] interpreted the distinct enlargement of the utricle in ariid catfishes—along with a unique structure of the macula utriculi that runs as a ribbon along the equatorial region—as a potential adaptation that facilitates low-frequency hearing. However, not all ariids possess a pronounced sensitivity to low frequencies [45]. Likewise, the tripartite macula utriculi in Clupeiformes that is connected to intracranial extensions of the swim bladder was hypothesized to play a role for ultrasound detection [46]. But although all clupeiform fishes show this modification, only representatives of the subfamily Alosinae seem to be able to detect ultrasound [47-50]. Altogether, these results highlight the need for neurophysiological experimentation in *E. maculatus* and other cichlids to test the potential role of the utricle in enhanced auditory abilities.

### Enlargement of macula regions and increased number of ciliary bundles in *E. maculatus*

Studies on sciaenids with anterior swim bladder extensions found an enlargement of the anterior region of the macula sacculi, the ostium [22,51,52]. We observed a similar expansion of the anterior part of the macula sacculi in *E. maculatus*, suggesting a functional relationship between this macula and enhanced auditory abilities as proposed for sciaenids [22]. Interestingly, *E. maculatus* also displays a dilatation of the anterior portion of the macula lagenae. The lagena in this species is in direct contact with the gas-filled part of the swim bladder extension [30]. Those two findings may be suggestive of a distinct role of both the saccule and the lagena for sound perception in *E. maculatus* and probably members of the Etroplinae in general.

Regarding the enlargement and strong 3D curvature of the anterior arm of the macula lagenae and the lacinia of the macula utriculi the question arises of whether this curvature evolved as a specific response to selection for improved physiological function, or if an altered curvature merely represents a by-product of the enlargement itself provoked by spatial constraints. Further growth of the anterior arm of the macula lagenae or the lacinia is not possible in vertical or lateral plane but has to follow the curvature of the walls of the end organs. Deng et al. [28] proposed a similar explanation for the unique bilobate shape of the anterior portion of the macula utriculi observed in the family Melamphaidae: the ampulla of the anterior semicircular canal may limit further growth of the anterior part of the macula utriculi in these fishes, while portions located to the left and to the right can expand, resulting in the characteristic macula contour outline. On the other hand, bringing ciliary bundles into a new direction and thus producing a wider range of ciliary bundle orientations by switching certain portions from 2D into a curved (i.e., 3D) shape might also be easier to accomplish than the formation of additional orientation groups on a flat macula, as pattern formation is a complex genetically directed process (e.g., [53,54]). We argue that identifying the “primary target of selection” (direct selection on altered orientation patterns or a pleiotropic effect due to the macula enlargement) is intrinsically difficult—if not impossible—as both modifications are tightly linked.

Assuming that the enlarged and strongly curved parts of the macula utriculi and macula lagenae in *E. maculatus* may account for improved vestibular and/or auditory abilities (sound source localization), the exact mode of stimulation of the hair cells on these macula portions still remains elusive, as they are not covered by the respective otolith. In areas overlain by an otolith, hair cells are stimulated by the ‘forth and back’ movement of the denser otolith (e.g., [55,56]). Otolith movement, in turn, is provoked by a sound source (sense of hearing) or by linear accelerations (vestibular sense) (e.g., [56]). It is therefore unlikely that the enlarged and curved lacinia of the macula utriculi or the anterior arm of the macula lagenae play a role in the improvement of the vestibular sense. For stimulation of uncovered macula areas, Rogers and Cox [57] and Rogers and Zeddies [58] proposed a model in which sound that is incident from a certain angle to the macula may lead to an ‘otolith-independent’ displacement of the macula and endolymphatic fluid again resulting in deflecting the ciliary bundles and thus stimulation of the hair cells. This may improve the signal-to-noise ratio and lower the detection thresholds [58]. Another possible way of stimulation was hypothesized by Popper [21], assuming that parts not covered by the otolith might be indirectly stimulated if the otolith movement was “transmitted” to the “uncovered” hair cells via the otolithic membrane.

The higher total number of ciliary bundles on the macula sacculi and macula lagenae in *E. maculatus* can be explained by the larger total area of these maculae compared to *H. guttatus* and especially *S. tinanti*[34,59]. Densities of ciliary bundles on the maculae, however, were partly lower or even lowest in *E. maculatus* (Table 2), which is at odds with predictions derived from the observation of enhanced auditory sensitivities. Higher ciliary bundle densities were found to be correlated with higher auditory sensitivities [33] or widening of the detectable frequency range in other teleosts [60]. Plainfin midshipman (*Porichthys notatus*, Batrachoididae) females, for example, show an increase in the range of frequency sensitivity during the breeding season [61,62], when ciliary bundle density on the macula sacculi is highest [33]. On the other hand, the effects of higher total number of ciliary bundles in *E. maculatus* are unclear because comparative studies that quantified ciliary bundles are generally rare and evaluated densities only (e.g., [22,52,60]). In the study by Webb et al. [24] total numbers of ciliary bundles are presented for chaetodontid fishes with and without anterior swim bladder extensions; however, sample sizes were small and showed considerable variation even within species. As we do not have a compelling explanation for the unexpectedly lower ciliary bundle densities found in *E. maculatus* at this point, this aspect needs further assessment.

### Interspecific differences of otolith size (area and weight)

*Etroplus maculatus* had slightly heavier saccular and distinctly heavier utricular otoliths which according to Lychakov and Rebane [63] can be interpreted in the context of enhanced sensitivities to auditory and/or vestibular stimuli. Following this line of argument, the large but very light (i.e., less dense) lagenar otolith in *E. maculatus* contradicts the interpretation that the lagena plays a major role in the enhanced auditory abilities due to its link to the swim bladder extension [30]. Lighter and less dense otoliths have been shown experimentally to negatively affect auditory sensitivities [36].

So, how can the light lagenar otoliths of *E. maculatus* be explained? One could argue that the lagena is not primarily involved in the improved auditory abilities despite the intimate swim bladder-lagena connection [30]. On the other hand, it is conceivable that there is not necessarily a simple and direct correlation with otolith mass or density. Although Ramcharitar et al. [27] found heavier saccular otoliths in weakfish (*Cynoscion regalis*, Sciaenidae)—a species displaying a close relationship between the swim bladder and the saccule—compared to a species lacking any swim bladder specializations (*Leiostomus xanthurus*), the authors did not find differences in auditory sensitivities between the two species. Schulz-Mirbach et al. [64] reported on distinct differences in otolith weight between cave- and surface-dwelling ecotypes of Atlantic mollies (*Poecilia mexicana*, Poeciliidae), but found strikingly similar auditory sensitivities. Finally, otophysans which show the most specialized swim bladder-inner ear connection via a chain of ossicles and ligaments and possess highly improved auditory capabilities (e.g., [17]), show saccular otoliths with a thin and fragile, needle-shaped appearance (e.g., [65]). A recent study by Krysl et al. [66] suggested a strong influence of otolith shape on otolith movement, highlighting the need for experimental studies on the effects of otolith dimensions like mass and shape on inner ear functions. In addition, the wings of the saccular otoliths in otophysans are in direct line with fluid flow [67,68] and thus the wings may help to move a light otolith better than a compact and dense one (pers. comm. A. N. Popper).

## Concluding remarks

Our study highlights the importance of comparing and interpreting orientation patterns of ciliary bundles in 3D. Until now, investigations of otolith vibration in the fish inner ear have only made use of CT data of otoliths [69]. Our detailed microCT-based 3D data of otoliths, maculae and the corresponding orientation patterns of ciliary bundles may thus be used in further studies to develop more complex models of otolith movement and the role of the swim bladder (including anterior extensions) as a sound pressure particle motion transducer using finite element modeling (FEM) and vibration analysis (e.g., [66,69,70]).

## Competing interests

The authors declare that they have no competing interests.

## Authors’ contributions

TSM, FL, MP, BM, and MH conceived the study. TSM carried out the preparations and analyses except microCT imaging (BM) and histological serial sectioning. MH and BM provided support with 3D reconstructions. MH created the interactive 3D models. All authors equally contributed to the writing of the manuscript. All authors read and approved the final manuscript.

## Supplementary Material

Additional file 1 **Interactive 3D model of the left inner ear of *Steatocranus tinanti*.** Cristae of the anterior (green), horizontal (yellow), and posterior (blue) semicircular canals; otoliths of the utricle (red), saccule (purple), and the lagena (yellow); maculae of the utricle (macula utriculi; light brown), saccule (macula sacculi; yellow orange), and the lagena (macula lagenae; dark brown / pink grey in 3D). Note projection of the 2D orientation patterns of ciliary bundles onto the 3D models of the macula lagenae, i.e. on the inner surface facing the lagenar otolith. The interactive 3D model can be accessed by clicking onto the figure (Adobe Reader Version 7 or higher required). Rotate model: drag with left mouse button pressed; shift model: same action + ctrl; zoom: use mouse wheel (or change default action for left mouse button). For selection (or changed transparency) of components use the model tree, switch between prefab views or change surface visualization (e.g. lighting, render mode, crop etc.). Deactivate 3D content via context menu (right mouse click). Click here for file

Additional file 2 **Interactive 3D model of the left inner ear of *Hemichromis guttatus*.** Cristae of the horizontal (yellow), and posterior (blue) semicircular canals; otoliths of the utricle (red), saccule (purple), and the lagena (yellow); maculae of the utricle (macula utriculi; light brown), saccule (macula sacculi; yellow orange), and the lagena (macula lagenae; dark brown). The interactive 3D model can be accessed by clicking onto the figure (Adobe Reader Version 7 or higher required). Rotate model: drag with left mouse button pressed; shift model: same action + ctrl; zoom: use mouse wheel (or change default action for left mouse button). For selection (or changed transparency) of components use the model tree, switch between prefab views or change surface visualization (e.g. lighting, render mode, crop etc.). Deactivate 3D content via context menu (right mouse click). Click here for file

Additional file 3 **Interactive 3D model of the left inner ear of *Etroplus maculatus*.** Cristae of the anterior (green), horizontal (yellow), and posterior (blue) semicircular canals; otoliths of the utricle (red), saccule (purple), and the lagena (yellow); maculae of the utricle (macula utriculi; light brown), saccule (macula sacculi; yellow orange), and the lagena (macula lagenae; dark brown, pink grey in 3D). Note projection of the 2D orientation patterns of ciliary bundles onto the 3D models of the macula lagenae, i.e. on the inner surface facing the lagenar otolith. The interactive 3D model can be accessed by clicking onto the figure (Adobe Reader Version 7 or higher required). Rotate model: drag with left mouse button pressed; shift model: same action + ctrl; zoom: use mouse wheel (or change default action for left mouse button). For selection (or changed transparency) of components use the model tree, switch between prefab views or change surface visualization (e.g. lighting, render mode, crop etc.). Deactivate 3D content via context menu (right mouse click). Click here for file

Additional file 4 **Projection of 2D orientation patterns onto the 3D models of the macula utriculi and macula lagenae.** Macula utriculi of *S. tinanti* (A), *H. guttatus* (B), and *E. maculatus* (C) and macula lagenae of *E. maculatus* (D) in posteromedial view. Especially the strongly curved lacinia (macula utriculi) and anterior arm (macula lagenae) in *E. maculatus* demonstrate the importance of orientation patterns of ciliary bundles shown in 3D. The interactive 3D model of the macula utriculi of *Etroplus* can be accessed by clicking onto the figure (Adobe Reader Version 7 or higher required). Rotate model: drag with left mouse button pressed; shift model: same action + ctrl; zoom: use mouse wheel (or change default action for left mouse button). For selection (or changed transparency) of components use the model tree, switch between prefab views or change surface visualization (e.g. lighting, render mode, crop etc.). Deactivate 3D content via context menu (right mouse click). Click here for file
