# Supplementary material for: Are accessory hearing structures linked to inner ear morphology? Insights from 3D orientation patterns of ciliary bundles in three cichlid species
Source: Front Zool. 2014 Mar 19;11:25. doi: 10.1186/1742-9994-11-25 (PMC3999956; doi:10.1186/1742-9994-11-25)
Supplement: Additional file 1 — Interactive 3D model of the left inner ear of Steatocranus tinanti. Cristae of the anterior (green), horizontal (yellow), and posterior (blue) semicircular canals; otoliths of the utricle (red), saccule (purple), and the lagena (yellow); maculae of the utricle (macula utriculi; light brown), saccule (macula sacculi; yellow orange), and the lagena (macula lagenae; dark brown / pink grey in 3D). Note projection of the 2D orientation patterns of ciliary bundles onto the 3D models of the macula lagenae, i.e. on the inner surface facing the lagenar otolith. The interactive 3D model can be accessed by clicking onto the figure (Adobe Reader Version 7 or higher required). Rotate model: drag with left mouse button pressed; shift model: same action + ctrl; zoom: use mouse wheel (or change default action for left mouse button). For selection (or changed transparency) of components use the model tree, switch between prefab views or change surface visualization (e.g. lighting, render mode, crop etc.). Deactivate 3D content via context menu (right mouse click). [file 1742-9994-11-25-S1.pdf]

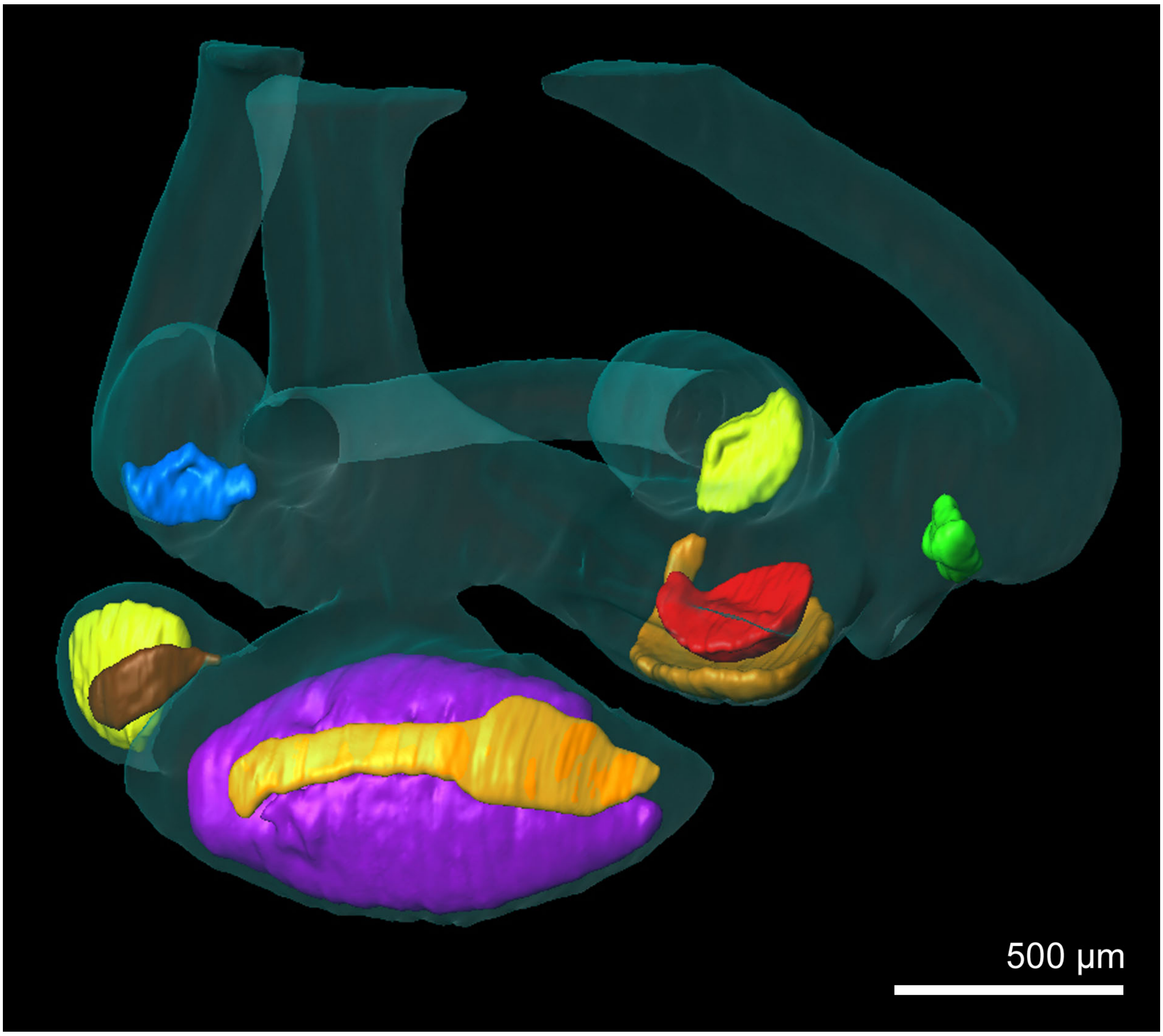

Additional file 1. Interactive 3D model of the left inner ear of *Steatocranus tinanti*.

Cristae of the anterior (green), horizontal (yellow), and posterior (blue) semicircular canals; otoliths of the utricle (red), saccule (purple), and the lagena (yellow); maculae of the utricle (macula utriculi; light brown), saccule (macula sacculi; yellow orange), and the lagena (macula lagenae; dark brown / pink grey in 3D). Note projection of the 2D orientation patterns of ciliary bundles onto the 3D models of the macula lagenae, i.e. on the inner surface facing the lagenar otolith. The **interactive 3D model** can be accessed by clicking onto the figure (Adobe Reader Version 7 or higher required). Rotate model: drag with left mouse button pressed; shift model: same action + ctrl; zoom: use mouse wheel (or change default action for left mouse button). For selection (or changed transparency) of components in the model tree, switch between prefab views or change surface visualization (e.g. lighting, render mode, crop etc.). Deactivate 3D content via context menu (right mouse click).
