# Supplementary material for: Are accessory hearing structures linked to inner ear morphology? Insights from 3D orientation patterns of ciliary bundles in three cichlid species
Source: Front Zool. 2014 Mar 19;11:25. doi: 10.1186/1742-9994-11-25 (PMC3999956; doi:10.1186/1742-9994-11-25)
Supplement: Additional file 4 — Projection of 2D orientation patterns onto the 3D models of the macula utriculi and macula lagenae. Macula utriculi of S. tinanti (A), H. guttatus (B), and E. maculatus (C) and macula lagenae of E. maculatus (D) in posteromedial view. Especially the strongly curved lacinia (macula utriculi) and anterior arm (macula lagenae) in E. maculatus demonstrate the importance of orientation patterns of ciliary bundles shown in 3D. The interactive 3D model of the macula utriculi of Etroplus can be accessed by clicking onto the figure (Adobe Reader Version 7 or higher required). Rotate model: drag with left mouse button pressed; shift model: same action + ctrl; zoom: use mouse wheel (or change default action for left mouse button). For selection (or changed transparency) of components use the model tree, switch between prefab views or change surface visualization (e.g. lighting, render mode, crop etc.). Deactivate 3D content via context menu (right mouse click). [file 1742-9994-11-25-S4.pdf]

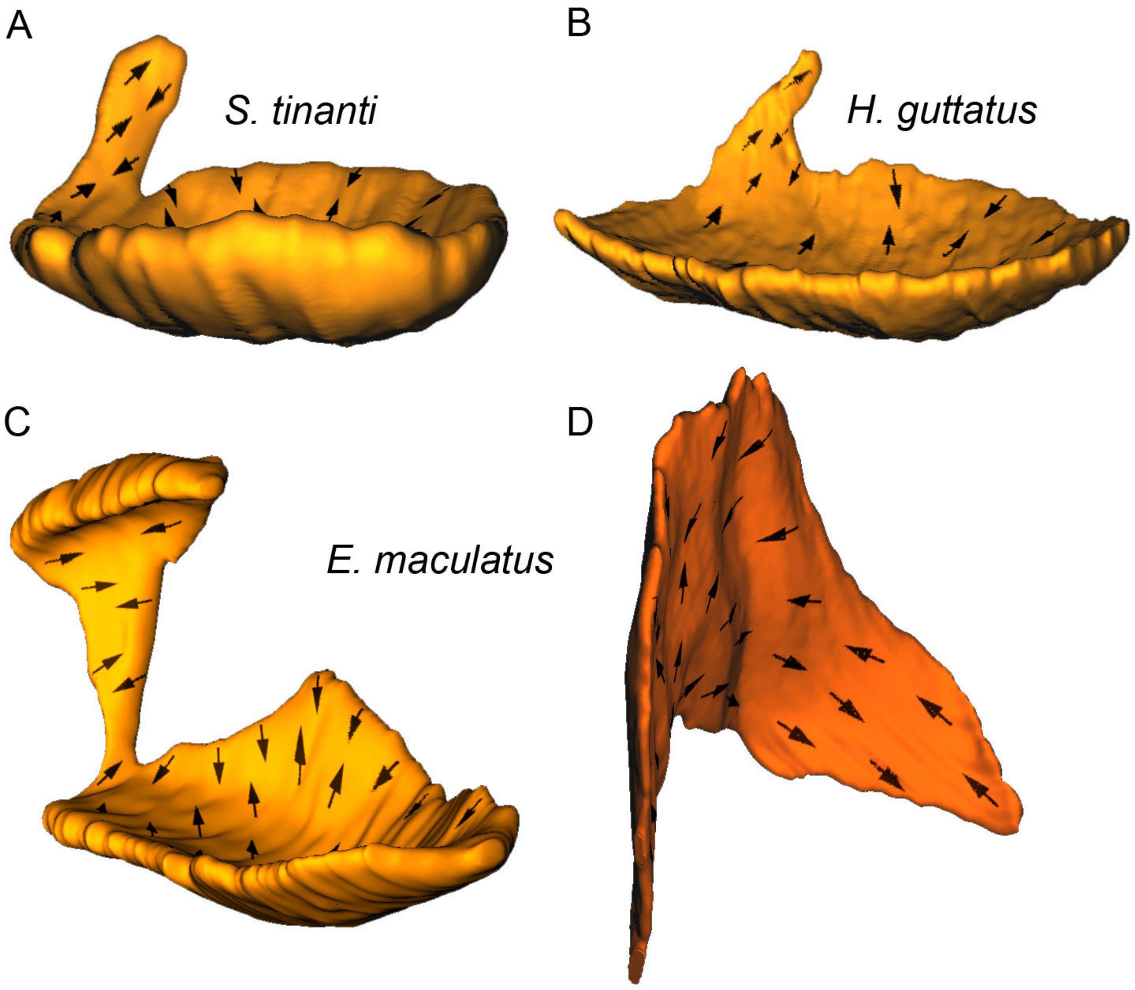

Additional file 4. Projection of 2D orientation patterns onto the 3D models of the macula utriculi and macula lagenae. Macula utriculi of *S. tinanti* (A), *H. guttatus* (B), and *E. maculatus* (C) and macula lagenae of *E. maculatus* (D) in posteromedial view. Especially the strongly curved lacinia (macula utriculi) and anterior arm (macula lagenae) in *E. maculatus* demonstrate the importance of orientation patterns of ciliary bundles shown in 3D. The **interactive 3D model of the macula utriculi of *Etroplus*** can be accessed by clicking onto the figure (Adobe Reader Version 7 or higher required). Rotate model: drag with left mouse button pressed; shift model: same action + ctrl; zoom: use mouse wheel (or change default action for left mouse button). For selection (or changed transparency) of components use the model tree, switch between prefab views or change surface visualization (e.g. lighting, render mode, crop etc.). Deactivate 3D content via context menu (right mouse click).
